# Prognosis Determinants after Cardioverter-Defibrillators Implantation in Brazil

**DOI:** 10.5935/abc.20170034

**Published:** 2017-03

**Authors:** Arn Migowski, Regina Maria de Aquino Xavier

**Affiliations:** Instituto Nacional de Cardiologia, Rio de Janeiro, RJ - Brazil

**Keywords:** Cardiac Resynchronization, Therapy Devices, Defibrillators, Implantable, Chagas Cardiomyopathy, Survival Analysis, Brazil

The study by Silva et al.^1^ presented the results of a series of cases from a single-center registry of implantable cardiac devices, including 28 implantations of isolated cardioverter defibrillators (ICD) and 14 associated to resynchronization (CRT-ICD), in addition to procedures for generator replacement and handling of electrode cable.^1^ ICD and CRT-ICD implantations were considered the strongest predictors of re-admission into the hospital during a six-month period after the surgical procedure, although it is not clear whether there was adjustment by functional class or left ventricular ejection fraction.^1^

We have recently published the results of short, medium, and long term follow-ups of all ICD and CRT-ICD implantations funded by The Brazilian Unified Healthcare System (SUS) in all of Brazil, between 2001 and 2007, including 3,295 ICD implantations in 85 hospitals and 681 CRT-ICD implantations in 50 hospitals.^2^

In our study, when compared to the ICD implantation group, patients who underwent CRT-ICD implantation had a worse mortality relative to the procedure and also a worse survival in the medium and long run.^2^ However, in the study by Silva et al.^1^, there apparently was no worse short-term prognosis of patients who underwent CRT-ICD with relation to those who underwent ICD implantation, at least in regards to re-admission.^1^ In relation to survival time, it would be interesting to consider stratification by device type, since the presented 6-month survival^1^ seemed low in relation to our finding,^2^ especially considering most implantations were for pacemakers.^1^ The percentage of double-chamber ICD would also be interesting information, considering some studies point to an association with poorer results.^2^

We have observed in the national multi-center study that the type of technique used for CRT-ICD implantation affected short-term mortality, and there was an important decrease in survival, approximately four years after the initial implantation in this group, possibly related to complications associated to the need for intervention.^2^ It would be interesting to know the magnitude of re-interventions in the short period of follow-up of the single-center registry and the possible impacts of implantation techniques in the outcomes.

The greater representativeness of Chagas heart disease, in the group with complications in the study by Silva et al.^1^, may be related to the type of implanted device. In our study, Chagas heart disease patients did not have a worse prognosis when compared to those with ischemic heart disease,^2^ similarly to another study recently published in *Arquivos Brasileiros de Cardiologia.*^3^
