# Supplementary material for: Prognosis Determinants after Cardioverter-Defibrillators Implantation in Brazil
Source: Arq Bras Cardiol. 2017 Mar;108(3):283–4. doi: 10.5935/abc.20170034 (PMC5389881; doi:10.5935/abc.20170034)
Supplement: Related texts

# Reply

**Authors:** Katia Regina da Silva, Caio Marcos de Moraes Albertini, Elizabeth Sartori Crevelari, Eduardo Infante Januzzi de Carvalho, Alfredo Inácio Fiorelli, Martino Martinelli Filho, Roberto Costa

The care with patients with implantable electronic cardiac devices (IECD) demands more and more the attention to the different clinical presentations of these patients. Such differences are becoming more prominent due to two main factors: an increase in the population longevity, which has increased comorbidities, and the increasing frequency of patients with cardiomyopathy and ventricular dysfunction.

We believe that the main message of this publication is to prove, through data derived from real clinical practice, that older patients, just as individuals with severe left ventricular dysfunction, are more prone to clinical events, related or not to cardiac disease, that require readmission to the hospital and that, therefore, must be observed more closely in order to be avoided.

Undoubtedly, ICD and CRT incorporation to the list of artificial cardiac stimulation procedures has brought a considerable contingent of patients with severe heart failure, which did not use to be as frequent when the only implanted devices were pacemakers. It is also not likely that this is the reason for the association mentioned in the commentary. With regards specifically to the relation between Chagas disease and mortality, our study was not designed with this objective nor would it have the sample power for that.

To make any comparison between the results of the presently published study and those of the publication of the comment's authors does not seem possible. While our study is a prospective analysis of primary data collected from a population of patients with all types of implantable devices, treated in one single, highly specialized, center, actively monitored by the researchers, the study by Migowski et al. is a retrospective analysis of secondary data obtained from administrative bases of SUS, from a population with cardio-defibrillators implanted at various centers with a varied level of specialization. Furthermore, the follow-up information is clearly incomplete, especially considering the authors of the study themselves have informed they censored all deaths from causes not related to heart disease or treatment.

We believe an attempt to make this kind of comparison would be like trying to compare the wine from a single producer to water from a container whose samples come from different sources.

**Katia Regina da Silva Caio Marcos de Moraes Albertini** **Elizabeth Sartori Crevelari** **Eduardo Infante Januzzi de Carvalho** **Alfredo Inácio Fiorelli** **Martino Martinelli Filho** **Roberto Costa**
